# Acute Prenatal Hypoxia in Rats Affects Physiology and Brain Metabolism in the Offspring, Dependent on Sex and Gestational Age

**DOI:** 10.3390/ijms23052579

**Published:** 2022-02-25

**Authors:** Anastasia V. Graf, Maria V. Maslova, Artem V. Artiukhov, Alexander L. Ksenofontov, Vasily A. Aleshin, Victoria I. Bunik

**Affiliations:** 1 Faculty of Biology, Lomonosov Moscow State University, 119234 Moscow, Russia; nastjushka@gmail.com (A.V.G.); maslova_masha@mail.ru (M.V.M.); 2 Department of Biokinetics, A. N. Belozersky Institute of Physicochemical Biology, Lomonosov Moscow State University, 119234 Moscow, Russia; whitelord_1994@mail.ru (A.V.A.); ksenofon@belozersky.msu.ru (A.L.K.); aleshin_vasily@mail.ru (V.A.A.); 3 Department of Biochemistry, Sechenov University, 119048 Moscow, Russia; 4 Faculty of Bioengineering and Bioinformatics, Lomonosov Moscow State University, 119234 Moscow, Russia

**Keywords:** brain neurotransmitter metabolism, malic enzyme, 2-oxoglutarate dehydrogenase, sex-specific effects of acute prenatal hypoxia

## Abstract

Hypoxia is damaging to the fetus, but the developmental impact may vary, with underlying molecular mechanisms unclear. We demonstrate the dependence of physiological and biochemical effects of acute prenatal hypoxia (APH) on sex and gestational age. Compared to control rats, APH on the 10th day of pregnancy (APH-10) increases locomotion in both the male and female offspring, additionally increasing exploratory activity and decreasing anxiety in the males. Compared to APH-10, APH on the 20th day of pregnancy (APH-20) induces less behavioral perturbations. ECG is changed similarly in all offspring only by APH-10. Sexual dimorphism in the APH outcome on behavior is also observed in the brain acetylation system and 2-oxoglutarate dehydrogenase reaction, essential for neurotransmitter metabolism. In view of the perturbed behavior, more biochemical parameters in the brains are assessed after APH-20. Of the six enzymes, APH-20 significantly decreases the malic enzyme activity in both sexes. Among 24 amino acids and dipeptides, APH-20 increases the levels of only three amino acids (Phe, Thr, and Trp) in male offspring, and of seven amino acids (Glu, Gly, Phe, Trp, Ser, Thr, Asn) and carnosine in the female offspring. Thus, a higher reactivity of the brain metabolism to APH stabilizes the behavior. The behavior and brain biochemistry demonstrate sexually dimorphic responses to APH at both gestational stages, whereas the APH effects on ECG depend on gestational age rather than sex.

## 1. Introduction

Physiological hypoxia is an important factor in embryonic development, essential for the proper formation of neural and cardiovascular systems [1,2,3]. However, excessive unphysiological hypoxia may lead to embryonic death or lasting negative consequences in offspring, such as developmental abnormalities and postnatal pathologies, including epilepsy, cerebral palsy, mental retardation, increased risks of arterial hypertension and cardiovascular diseases [4,5,6,7]. Although acute hypoxia during pregnancy (APH) occurs less frequently than chronic hypoxia, the consequences of short-term episodes of acute hypoxia, usually lasting a few minutes, may be even more serious than those of chronic hypoxia, although the developmental impact is highly variable in both cases [8,9,10].

In the clinic, short-term episodes of acute hypoxia occur most often during labour and delivery, associated with uterine contractions and/or compressions of the umbilical cord [10]. Hence, most studies of fetal hypoxia focus on the period of late gestation. Although scarce, the information on the impact of acute hypoxia in the earlier periods is available too [11,12]. The periods of organogenesis, labour and delivery represent typical examples of critical periods of ontogenesis [13,14,15]. Presumably, during the period of intense growth and/or essential physiological changes, the foetus tissues and organs are most susceptible to different insults, as biological programs of development are especially prone to perturbations. The consequences of insults occurring at such stages usually appear after a period of latency, manifested spontaneously, or provoked by some other insults [16]. However, the question on the role of gestational age at the time of hypoxic exposure has not been extensively addressed in the literature [12,17,18].

Ontogenetic disturbances induced by prenatal hypoxia are known to manifest in hyperactivity and/or learning/memory deficits, including attention deficit combined with anxiety [19,20]. The impact of prenatal stress on fetal brain development may also be expressed in the changed ECG parameters in offspring, that correlate with the newborn’s stress and stress-related behaviors [21,22,23,24]. Serious long-term effects of prenatal hypoxia on the cardiovascular system link prenatal stress to increased risk of cardiovascular disease in adults [16,25,26]. Prenatal hypoxia has been associated with damage to the brain regions which regulate behavior and emotions [27]. The parameters of ECG and behavior may therefore be useful for early diagnosis of the postnatal heart and neurological pathologies [28]. It is worth noting that clinical and experimental studies indicate that there are sex differences in the sensitivity to gestational stress [11,16,29,30,31].

The goal of our current study is to assess the significance of gestational age and sex for physiological and molecular consequences of prenatal hypoxic exposure. Pregnant rats are exposed to acute hypoxia during the early organogenesis period, i.e., on day 10 in utero (APH-10), or shortly before the delivery, i.e., on day 20 in utero (APH-20). The chosen time points of rat development correspond to the third and 13th–15th weeks of pregnancy in human development, respectively [15,32]. In our study, physiological effects are assessed in the male and female offspring, using behavioral tests and non-invasive ECG. Simultaneous assays of the acetylation system, proteins, and metabolites in the offspring brain are employed to decipher biochemical mechanisms of the dimorphic impact of prenatal hypoxia on physiology. Using these biochemical indicators, we show significant metabolic changes in the hypoxia-affected brain of offspring and analyze their relationship to the physiological changes.

## 2. Results

### 2.1. Behavioral Parameters Are Affected by APH, Dependent on the Offspring Sex and Gestational Age at the Insult Exposure

**Figure 1:**
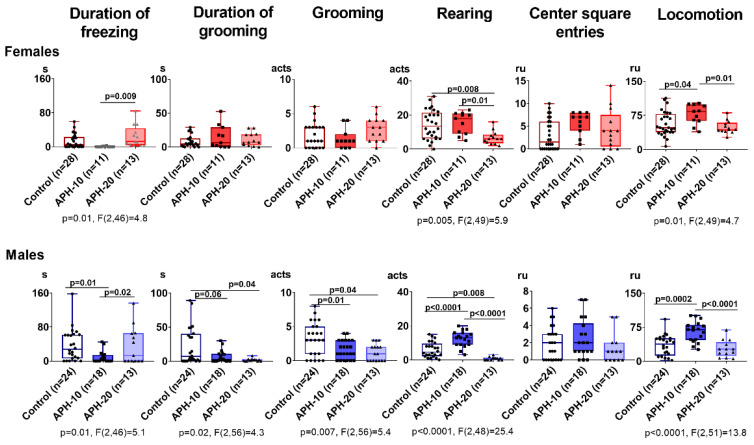
Influence of acute prenatal hypoxia on behavioral parameters of the adult female and male offspring. Behavioral parameters indicated above the graphs are presented as mean ± SEM, expressed in seconds (s) (duration of freezing and grooming), acts (number of grooming, rearing) or relative units (ru) (center square entries, locomotion). Significance of the differences between the two indicated groups is shown on the graphs as *p*-values, estimated by using one-way ANOVA with Tukey’s post hoc test. For the parameters with significant (*p* < 0.05) differences, the *p*-values and F-statistics of one-way ANOVA are shown below the graphs. *n*—the number of rats in each group.

Figure 1 demonstrates that APH-exposed offspring exhibit altered behavioral patterns vs control animals, with the alterations depending on sex and gestational age of the exposure. In both the male and female offspring, the APH in the period critical for organogenesis, i.e., on the 10th day of gestation (APH-10), increases locomotion, compared to the control group. In males, APH-10 additionally elevates rearing and reduces grooming and its duration, indicative of a higher exploratory activity and a lower anxiety in the open field test. Overall, five out of the six estimated behavioral parameters are changed by APH-10 in the male offspring while only one is changed in the female offspring. Thus, APH-10 affects behavior more in the male than female offspring.

The APH on the 20th day of pregnancy (APH-20), i.e., in the period preceding the delivery, results in different and less pronounced behavioral perturbations in the offspring, compared to APH-10 (Figure 1). Both the male and female offspring exposed to APH-20, manifest decreased rearing, which in the males is added by decreases in grooming acts and their duration. Overall, three out of the six estimated behavioral parameters are changed by APH-20 in the male offspring, while only one is changed in the female offspring. Thus, behavioral reactivity to APH-20 is lower and different than that to APH-10, yet in both cases, the behavior is more affected in the male vs female offspring.

### 2.2. Only APH-10 Affects ECG, with the ECG Changes Similar in the Female and Male Offspring

**Figure 2:**
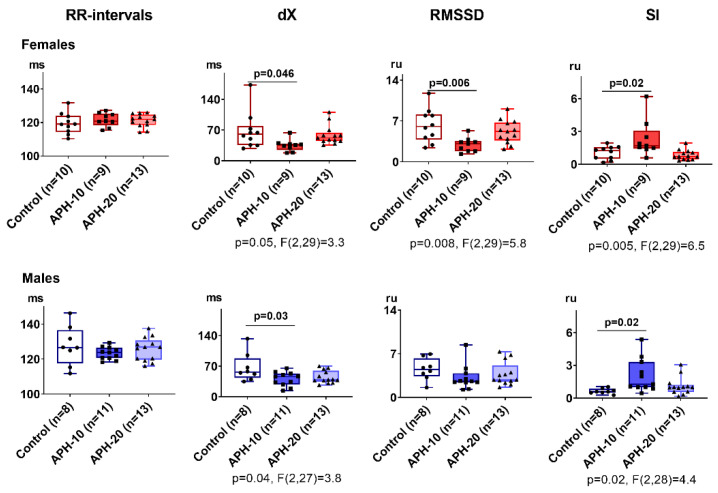
Influence of acute prenatal hypoxia on ECG parameters in the adult female and male offspring. ECG parameters are presented as the mean ± SEM, expressed in milliseconds (ms) or relative units (ru). Significances of the differences between the two groups are shown on the graphs as *p*-values estimated using one-way ANOVA with Tukey’s post hoc test. For the parameters with significant (*p* < 0.05) differences, the *p*-values and F-statistics of one-way ANOVA are shown below the graphs. *n*—the number of rats in each group.

Figure 2 demonstrates the influence of APH-10 and APH-20 on the parameters of non-invasive ECG. The presented changes indicate that, unlike the behavioral parameters, ECG is changed similarly in both the male and female offspring and only after APH-10. In both sexes, APH-10 reduces dX and increases the stress index (SI). Changes in RMSSD oppose those in SI, but the RMSSD decrease is of statistical significance in female offspring only.

No effects of APH-20 on ECG are observed. Thus, hypoxic exposure at the period critical for organogenesis affects ECG parameters of the offspring independent of sex.

### 2.3. Levels of Acetylation of 13–17 kDa Proteins and Expression of Mitochondrial Sirtuin 3 in the Offspring Brain Depend on the Offspring Sex and Gestational Age during the Acute Prenatal Hypoxia

**Figure 3:**
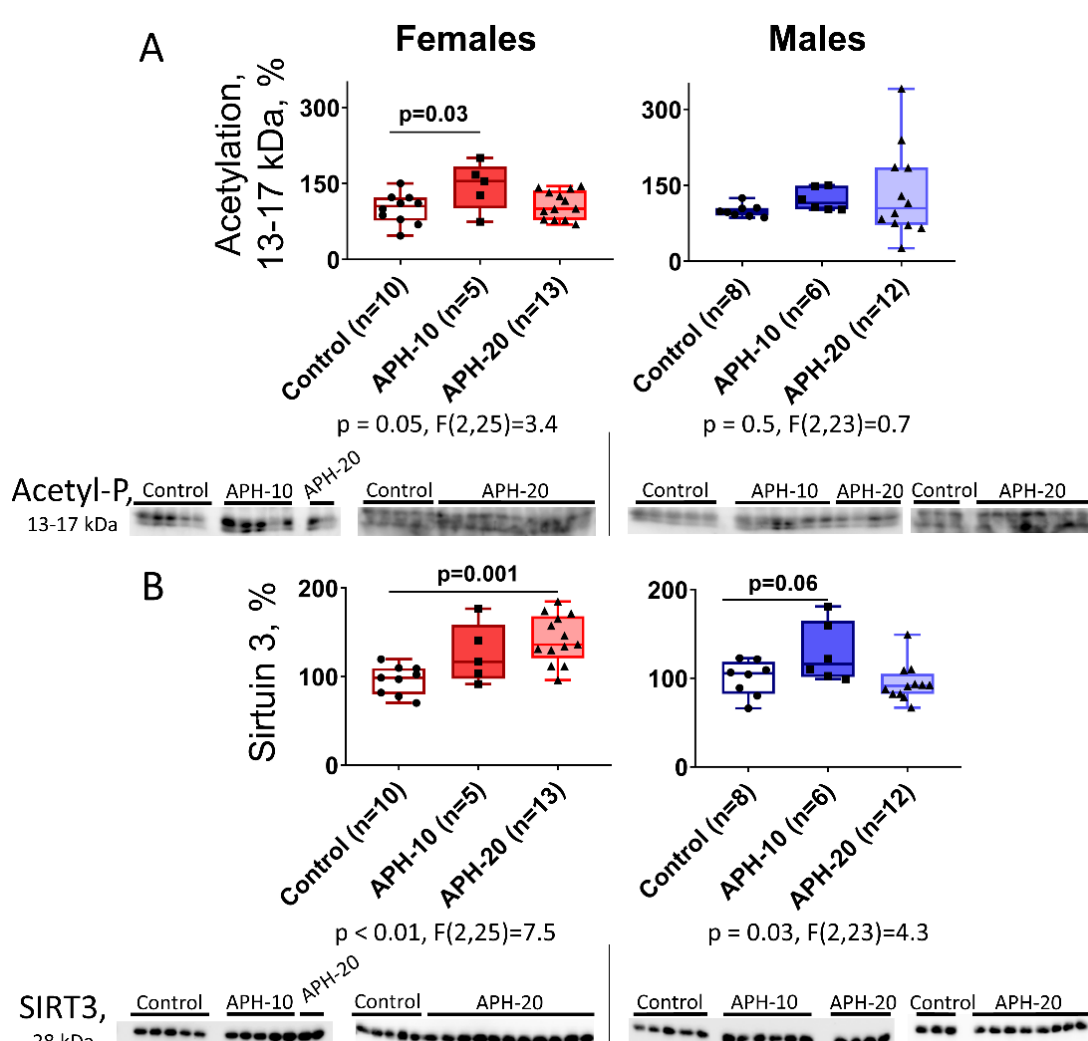
Influence of acute prenatal hypoxia at different gestational age on the levels of acetylation of low molecular mass proteins (acetyl-P) (**A**) and expression of sirtuin 3 (SIRT3) (**B**) in the brain of the APH-exposed female and male offspring. The *p*-values and F-statistics of one-way ANOVA are shown below the graphs. The results of the Tukey’s post hoc test are indicated on the graphs. The presented Western blots cover all rats of the analyzed sample. *n*—the number of rats in each group.

Long-term changes in behavior and ECG of the offspring exposed to prenatal hypoxia (Figure 1 and Figure 2) suggest epigenetic changes in the brain. We, therefore, used immunoblotting with antibodies specific to the acetylated proteins to assess the acetylation level of the immunoreactive protein bands within a range of apparent molecular masses, corresponding to those inherent in histones (13–17 kDa). As seen from Figure 3A, acetylation of these proteins is increased by APH-10 in the female offspring, while in the males no significant increase is observed.

As hypoxia primarily affects mitochondrial metabolism, we also checked the expression of mitochondrial sirtuin 3 in the brains of affected offspring. As seen from Figure 3B, the expression of sirtuin 3 exhibits a pattern, complementary to that of the acetylation of proteins of 13–17 kDa. When sirtuin 3 is increased, the acetylation of proteins of 13–17 kDa does not change. This occurs in female offspring exposed to APH-20, and in male offspring exposed to APH-10. However, when sirtuin 3 is not increased, the acetylation of proteins of 13–17 kDa does. This is obvious in female offspring exposed to APH-10.

### 2.4. Effects of APH on the Activity of OGDHC Critical for the Neurotransmitter Metabolism, in Cerebral Cortex of Offspring

**Figure 4:**
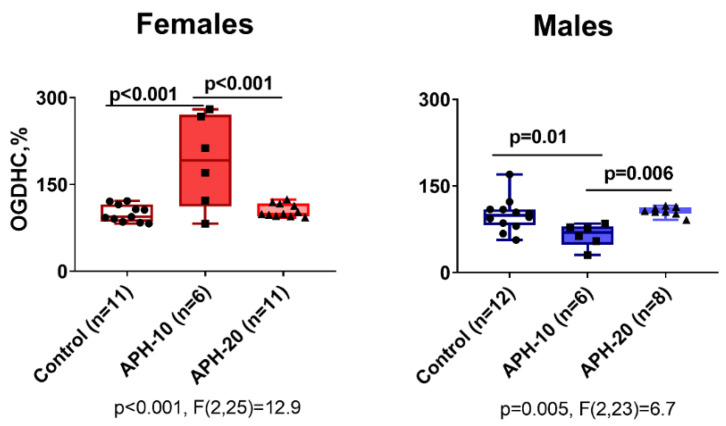
Influence of acute prenatal hypoxia on the activity of OGDHC in the brain cortex of the female and male offspring exposed to APH on the 10th or 20th day of gestation. Activities in the brain cortex of the adult female or male offspring are presented as the means ± SEM. The *p*-values on the graphs indicate significance of the differences between the groups, estimated by using one-way ANOVA with Tukey post hoc test. For the activities with significant (*p* < 0.05) differences the *p*-values and F-statistics of one-way ANOVA are shown below the graphs. *n*—the number of rats in each group.

Functional expression of 2-oxoglutarate dehydrogenase complex (OGDHC) limits the substrate flux through the TCA cycle in the brain mitochondria [33], thus determining the overall flux of the protein acetylating agent acetyl-CoA and the amino acid neurotransmitters metabolism. As shown in Figure 4, the activity of OGDHC in the offspring brain responds to APH depending on the offspring sex and gestational age of APH. That is, the changes in the OGDHC activity are opposite in the male and female offspring, observed after APH-10 only (Figure 4).

### 2.5. Alterations in Amino Acids, Dipeptides and Enzymes in the Cerebral Cortex of the Offspring Exposed to APH-20

**Figure 5:**
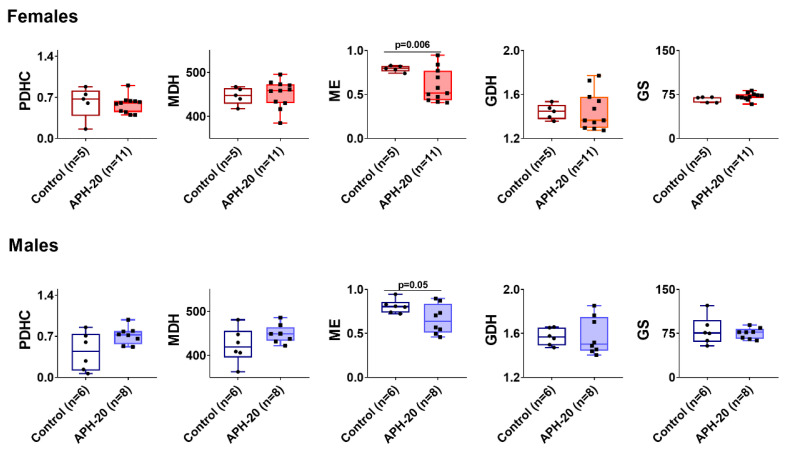
Influence of acute prenatal hypoxia on the 20th day of gestation (APH-20) on cerebral cortex enzymes in the male and female offspring of rats. The activities (micromole/min per g of tissue) are presented as the means ± SEM. *p*-values shown on the graphs, are determined using unpaired *t*-test with Welch’s correction. PDHC—pyruvate dehydrogenase complex, MDH—malate dehydrogenase, ME—malic enzyme, GDH—glutamate dehydrogenase, GS—glutamine synthase. *n*—The number of rats in each group.

The less affected behavior in offspring exposed to APH-20 than in those after APH-10, added by ECG changes after APH-10 only (Figure 1 and Figure 2), corresponds well to the fewer changes in the brain biochemical parameters after APH-10, compared to those after APH-20 (Figure 3 and Figure 4). Nevertheless, APH-20 does induce behavioral changes (Figure 1) along with the changed expression of mitochondrial sirtuin 3 in the brain (Figure 3B), with both the physiological and biochemical consequences of APH-20 exhibiting sexual dimorphism. In view of the changed expression of mitochondrial sirtuin 3 in the brain of female offspring after APH-20, some of the TCA cycle and associated enzymes, including those important for the acetyl-CoA metabolism and/or undergoing the sirtuin-3-dependent acetylation, have been assayed. Figure 5 demonstrates that the brain malic enzyme activity may serve as an enzymatic indicator of the APH-induced perturbations in both the female and male offspring.

Furthermore, the brain amino acid profiles, which are sensitive indicators of the brain neurotransmitter metabolism linker to behavior [34,35,36], have been assessed. From 24 amino acids and dipeptides quantified, the levels of Glu, Gly, Phe, Trp, Ser, Tyr, Asn, and carnosine increase in the cerebral cortex of female offspring, subjected to APH-20, while only those of Phe, Thr, and Trp increase in the APH-20-exposed males (Table 1). Thus, the metabolic profiles presented in Table 1 indicate that the amino acids metabolism of the APH-20-exposed offspring is changed less in males than females.

## 3. Discussion

In both animals and humans, the nervous system develops over a long time extending from the embryonic period through puberty [37]. In this work, we show how acute prenatal hypoxia perturbs normal development, leading to changes in the offspring behavior, ECG, and brain biochemistry. Our data indicate that the changes are strongly dependent on gestational age at the time of the insult. Changes in cardiac rhythm variability estimated by SI and RMSSD indices of ECG demonstrate a perturbed balance of sympathetic and parasympathetic activity [38] in the offspring exposed to APH-10. Although ECG is changed similarly in both the female and male offspring exposed to APH-10, RMSSD shows a statistically significant decrease in the females only. Increased SI in the APH-10 offspring corresponds to elevated sympathetic excitation, while decreased RMSSD in the female offspring additionally manifests significantly diminished parasympathetic activity. This phenotype strongly associates with cardiovascular disease [9,39,40], potentially related to the APH-induced reprogramming of several genes playing an important role in an increased susceptibility of the adult heart to ischemia [16]. Several studies suggest a correlation between reduced RMSSD and neurological symptoms such as apathy, withdrawal, and delusions [41], linking psychotic symptoms to autonomic cardiac dysfunction [42,43]. Hence, integrating parasympathetic inhibition and sympathetic activation, that determine adaptive response to external and internal changes [44], the changed ECG parameters mark negative effects of APH-10 on the adaptation in both sexes.

The heart rate variability, characterized by the estimated ECG parameters, is known to be a valuable non-invasive test to investigate the development and function of the autonomic nervous system. This system regulates cognitive and emotional development, along with the ability of an organism to cope with stressors [28,45,46,47,48]. Supporting this view, a subtle difference between the sexes, expressed in the significant decrease in RMSSD in the females only, is accompanied by a much more pronounced sexual dimorphism in the behavioral and biochemical parameters. A higher, compared to ECG, sensitivity of the behavioral and biochemical indicators of APH reveals significant sex dependence of the APH developmental consequences. However, in all the applied tests, i.e., ECG, behavior, and biochemistry, APH occurring in the period critical for organogenesis (APH-10) induces more consequences than APH shortly before delivery (APH-20).

The activity of the brain cortex OGDHC is known to correlate with the ECG parameters, the correlation mediated by the levels of the brain glutamate [49]. In the current work, we also observe that the APH-induced changes in the brain OGDHC level show a good correspondence to the changes in the offspring ECG. Interestingly, the opposite effects of APH-10 on the brain OGDHC activity in the female (an increase) and male (a decrease) offspring may underlie the sexual dimorphism in the reactivity of RMSSD to APH-10.

The 2-oxoglutarate/glutamate ratio, dependent on the OGDHC function, is of a signaling role in metabolic adaptations of various cell types [33], and an alteration of this ratio may be one of the factors triggering epigenetic changes. The latter may involve the rate-limiting role of OGDHC in the flux through the TCA cycle in the brain mitochondria [33,50], linking the 2-oxoglutarate/glutamate ratio to the metabolism of acetyl-CoA, which participates in the acetylation of proteins including histones. Our study points to an interesting correlation between the sexually dimorphic changes in the OGDHC activity (Figure 4) and the acetylation of proteins of 13–17 kDa (Figure 3A). In females, APH-10 increases the acetylation along with the OGDHC activity, while in males, none of the increases are observed. A higher flux through OGDHC in the female brains may manifest increased production of the TCA cycle substrate, acetyl-CoA. The increased acetyl-CoA levels are supported by a concomitant increase in the protein acetylation in the female brain. Changes of another component of the brain acetylation system, the protein deacetylase sirtuin 3, are complementary to those in the OGDHC activity and acetylation of proteins of 13–17 kDa. That is, sirtuin 3 appears to have a heightened control of the protein acetylation by acetyl-CoA when the flux through OGDHC is not increased. Indeed, under such settings, i.e., when the brain sirtuin 3 expression rises instead of the OGDHC activity, no increase in the acetylation of proteins of 13–17 kDa is observed, either in the female brain after APH-20, or in the male brain after APH-10.

Since OGDHC catalyzes a rate-limiting step in the TCA cycle [33], OGDHC function is also important for the TCA-cycle-associated amino acid metabolism which is of special significance for the brain neurotransmitters [36,51]. In particular, the brain glutamate levels mediate the link between brain OGDHC activity and ECG or behavioral parameters [36,49,52]. Moreover, we have recently shown that specific inhibition of OGDHC models the action of APH on the amino acid metabolism, with both actions changing accordingly due to the pregnancy [34,35]. Hence, in addition to the role in the protein acetylation, the opposite changes in brain OGDHC in the male and female offspring exposed to APH-10 may underlie the sex-dependent behavioral changes observed in these offspring.

Levels of brain amino acids are also sensitive indicators of the metabolic changes which are not deducible from the enzymatic activities assayed in vitro. Unlike the latter activities, in vivo fluxes through the enzymes are strongly dependent not only on the expression of functional enzymes but also on the steady-state concentrations of the enzyme substrates and cofactors, which are usually different from those of the in vitro assays. Hence, even when the enzymatic assays do not show significant changes in functional expression of OGDHC or affiliated enzymes, as in the case of the offspring exposed to APH-20, the steady-state concentrations of metabolites may change significantly.

Analysis of our current results reveals an interesting relationship between the levels of phenylalanine and tryptophane and behavior. The two amino acids are precursors of dopamine and serotonin. Their increased levels in both the female and male offspring, exposed to APH-20, may well underlie decreases in rearing, inherent in both sexes (Figure 1). However, multiple changes in the levels of other amino acids and carnosine after APH-20 are observed in female offspring only. In particular, the female brain demonstrates elevations in the major excitatory neurotransmitter glutamate and inhibitory neurotransmitter glycine. Concomitantly, the females exhibit no changes in the anxiety indicators, such as grooming and its duration, which are decreased in the APH-20-exposed males. Overall, one may conclude on the opposite expression of the biochemical and behavioral changes, resulting from the APH-20 exposure. In the male offspring, the biochemical markers, such as the activities or expression of the brain enzymes and metabolites, are affected less than in the female offspring. Among the amino acids, only the levels of phenylalanine, threonine, and tryptophan are increased in the male brain, while the levels of four additional amino acids (asparagine, glutamate, glycine, serine) and carnosine are increased in the female brain. This coincides with the change in the brain malic enzyme activity that is more pronounced in females (*p* = 0.006) than in males (*p* = 0.05). In contrast to the male brain, the female brain also demonstrates the effects of APH-20 on the protein deacetylase sirtuin 3, which deacetylates, in particular, glutamate dehydrogenase, whose function is strongly dependent on multiple acetylation sites [53]. Apart from the changes in the amino acid homeostasis due to changed expression of the malic enzyme activity and sirtuin 3, shown in our current work, the APH-20-perturbed amino acid levels may rely on a strong link between the amino acid availability and autophagy and mTOR signaling, both known to be affected by different hypoxic conditions in different tissues [54,55,56]. For instance, hippocampal neurogenesis is affected by chronic hypoxia through attenuation of the mTOR pathway [57]. In the fetal lung, gestational hypoxia also dysregulates mTORC1 activation, which perturbs vascular development, setting the stage for pulmonary vascular disease in adulthood [58]. Autophagosomes and autolysosomes are increased by hypoxia in fetal kidneys, where hypoxia perturbs fetal renal development through renal apoptosis and autophagy mediated by beclin 1 [59]. Hypoxia-inducible factor HIF-1a is also known to be involved with amino acid metabolism in ischemic brains of newborn pigs, where the levels of mitochondrial glutamic oxaloacetic transaminase GOT2 and glutamate-cysteine ligase correlate with the levels of HIF-1a [60]. Similar to our results (Table 1), the hypoxic insult in the newborn pigs also results in elevated glutamate. Thus, hypoxia-induced changes in the regulation of autophagy, mTOR1, and HIF1a signaling may contribute to the changed brain levels of amino acids in the APH-20-exposed offspring (Table 1).

Remarkably, both sexes demonstrate more of the delayed effects when the acute hypoxia occurs in the period critical for organogenesis, compared to the period shortly before the delivery. However, we show that a higher plasticity of the brain metabolism in the female vs male offspring is linked to a higher resistance of the female vs male behavior to the delayed effects of APH, observed in this work. Yet the APH-changed homeostasis of amino acids in the female brain (Table 1) may induce other pathologies in further life. The elevated levels of the brain neurotransmitters and neuromodulators (glutamate, aspartate, glycine) and their precursors (tryptophan, phenylalanine) in the APH-20-exposed offspring brain are worth noting in this regard. Increased secretion of monoamines, firstly, serotonin, during a critical perinatal period is known to cause emotional deficits in adulthood, affecting social interactions or resulting in anxiety-like and depressive-like behaviors [61]. Increased levels of brain glutamate are known to cause brain dysfunction in Alzheimer’s disease, bipolar disorder, and major depression, also being an early indicator of these diseases [62,63]. Glycine and serine modulate the activity of the glutamate-dependent NMDA receptors; furthermore, also the elevation of these amino acids in the offspring brain may increase the risk of neuropathologies. In particular, high levels of glycine and serine may cause seizures symptoms [64,65,66]

As a result, our data indicate that the biochemical effects of APH-20 are directed to maintaining behavioral patterns. Indeed, the former are more pronounced in the female than male offspring, while the latter change more in the male than female offspring. Thus, the multiple biochemical changes in the APH-20-exposed female brain are of a compensatory nature. Earlier, we have observed similar features, i.e., multiple biochemical changes accompanied by a better physiological stabilization, when comparing the reactivity to hypoxia or its chemical mimic of non-pregnant and pregnant female rats [34,35].

Our results support the hypothesis that prenatal hypoxia induces reprogramming of the nervous and cardiovascular system development. Both clinical and animal studies have suggested a close relationship between prenatal stress and the sexually dimorphic development of the most common diseases of the modern world later in life. Our work reveals that metabolic markers may be valuable predictors of the changes induced by prenatal hypoxia, enabling timely corrections to prevent postnatal disorders.

## 4. Materials and Methods

### 4.1. Animal Experiments

All animal experiments were performed according to the Guide for the Care and Use of Laboratory Animals published by the European Union Directives 86/609/EEC and 2010/63/EU and were approved by the Bioethics Committee of Lomonosov Moscow State University (protocol number 69-o from 9 June 2016). Animals were kept at 21 ± 2 °C and relative humidity 53 ± 5% with the 12/12 h light/dark cycle (lights on 9:00 = ZT 0, lights off 21:00 = ZT 12).

Wistar female rats about 270 ± 20 g were used in the experiment (*n* = 11). The rats were purchased from the State Research Center of the Russian Federation—Institute for Biomedical Problems, Russian Academy of Sciences, and were kept at our conditions for two weeks prior to the experiments. T/4K cages (555/4K, 580 × 375 × 200 mm) were used. Standard rodent pellet food (laboratorkorm.ru) and tap water were available ad libitum. Animals were kept by 56 in each cage. To obtain pregnant rats, two virgin female rats were located in a cage with one male. After 24 h, vaginal smears were examined. When sperm was found in the vaginal smear, it was considered as the 1st day of pregnancy, and the male rat was separated. A week before the delivery, the female rats were placed in individual T/3K cages (460 × 300 × 160 mm).

Pregnant rats were exposed to hypoxia as described previously [35,67] on the 10th or 20th day of pregnancy. These time points roughly correspond to the first and second trimester of human pregnancy. We did not notice a significant difference in the number of pups (9 ± 1) between the normal and hypoxic rats, although independent long-term physiological monitoring indicated that the pregnancy failures occurred more often in the latter vs former rats [68].

The offspring remained with the mothers until 28 days of age and were fed a standard diet till adulthood. The animals were housed in experimental groups of the same sex until the 40th day of life by 5–6 rats per T/4K cage. Cages were changed once a week. Long-term consequences of acute prenatal hypoxia were analyzed in the three months old adult offspring of both sexes.

The total number of animals in an experimental group, *n*, is indicated in the figures and tables.

### 4.2. Acute Hypobaric Hypoxia

Female rats were exposed to acute hypobaric hypoxia at 5% O_2_ (11,500 m altitude, 145 mm Hg) in a decompression chamber “Mez Mohelnice”(Mohelnice, Praha, Czech Republic) of 3.3 L volume, as described previously [35,68,69]. Pregnant rats exhibited a significant decrease in resistance to hypoxia, compared to the non-pregnant rats [52,67]. Hypoxic tolerance time was 169.6 ± 53.8 s and 148.3 ± 23.9 s on the 10th or 20th day of pregnancy, respectively.

### 4.3. Behavioral Parameters

The habituation response of the animals placed in a new environment was estimated in adult offspring (12 weeks old) of both sexes in the open field test (OpenScience, Moscow, Russia) [70]. The testing apparatus consisted of a circular polypropylene arena, with its floor divided into 19 sectors for visual scoring of animal locomotor activity. Each animal was placed in the center, and its behavior was recorded in complete silence under the light of a 15 W for 3 min. The number of grooming, time of grooming and freezing, number of rearing, the total numbers of squares (lines) crossed were recorded during this time. The employed behavioral parameters characterize anxiety, exploratory and locomotor activities as described previously [71,72].

### 4.4. Animal Electrocardiography (ECG)

ECG was registered using a noninvasive method on rats of free behavior as described earlier [66]. Autonomic control, representing relative contributions of the sympathetic and parasympathetic components of the nervous system activity, was assessed by the following parameters of ECG: the average RR interval in a sample, ms; dX—range of RR-intervals in a sample (RRmax−RRmin), ms; RMSSD—parasympathetic, or relaxation, index of the state of the nervous system (calculated as the square root of the mean of the sum of the squares of differences between adjacent RR-intervals); SI—sympathetic, or stress, index of the nervous systems state (calculated as the total number of all RR intervals divided by the height of the histogram of all RR intervals measured on a discrete scale with bins Equation (1)), [49,73].

(1) $$SI=\frac{\mathrm{AMo}}{2\times \mathrm{Mo}\times \Delta X}$$

After registration of behavior and ECG, the rats were sacrificed by decapitation using a guillotine (OpenScience, Russia). Decapitation is considered one of the least stressful and fastest methods to kill animals, which does not chemically modify brain tissue. The procedure was carried out according to accepted protocols [74] as described previously [72].

### 4.5. Enzyme Assays

Homogenization of the brain cortex and assays of the overall OGDHC activity of brain homogenates were performed as described earlier [51,52,67]. Cerebral cortex tissue was homogenized in 2.5 mL/1g of homogenization buffer (50 mM MOPS, pH = 7.0, containing 2.7 mM EDTA, 20% glycerol and protease inhibitors: 0,2 mM AEBSF, 0.16 µM aprotinin, 3.33 µM bestatin, 3 µM E-64, 2 µM leupeptin, and 1.4 µM pepstatin A). The homogenates were solubilized by sonication in Bioruptor (Diagenode, Liege, Belgium) and diluted with 40 mM Tris-HCl, pH = 7.4, containing 600 mM NaCl, 4 mM EDTA, 1% sodium deoxycholate, and 4% NP-40 at a ratio of 3:1. Activities of OGDHC, glutamate dehydrogenase, malate dehydrogenase, and malic enzyme were measured via spectrophotometric assay, detecting NADH or NADPH absorbance change at 340 nm. Pyruvate dehydrogenase complex was assayed spectrophotometrically using a coupled reaction between the reaction product, NADH, and iodonitrotetrazolium, detecting absorbance change at 500 nm [75,76]. Glutamine synthase was assayed by endpoint method, measuring the absorbance of its side reaction product, γ-gutamyl hydroxamate-Fe^3+^ complex in acidic condition at 540 nm [77].

### 4.6. Assay of Protein Lysine Acetylation and Sirtuin 3 by Western Blotting

The crude cerebral homogenates were diluted 20-fold in the sample buffer (60 mM Tris-HCl pH= 6.8, 10% glycerol, 2% SDS, 5% β-mercaptoethanol, 0.01% bromophenol blue), mixed gently, and incubated at 95℃ for 5 min. The levels of protein lysine acetylation in the homogenates were assayed using anti-acetyl-lysine primary antibody (Cell Signaling Technology, #9814). The levels of sirtuin 3 were assayed using the corresponding primary antibody (Cell Signaling Technology, #5490). Normalization of the protein acetylation or sirtuin 3 levels to the total protein in the corresponding gel lanes was performed, using the fluorescent quantification of the total protein in each lane [78]. Briefly, after electrophoresis, the gel with 2,2,2-trichloroethanol was UV-irradiated, and fluorescence was detected using the ChemiDoc MP imaging system (Bio-Rad, Hercules CA, USA). After the transfer of the proteins to the PVDF membrane, the membrane was blocked by the 5% BSA solution in Tris-buffered saline containing 0.1% Tween 20 (TBST) for 30 min at room temperature. The 1:2000 dilution of the primary antibodies (Cell Signaling Technology, #5490 and #9814) in TBST with 0.5% BSA and 1:3000 dilution of the corresponding secondary antibodies (Cell Signaling Technology, #7074) were used. Statistical analysis of gels and blots was performed in Image Lab 6.1 (Bio-Rad, Hercules, CA, USA).

### 4.7. Ninhydrine Quantification of Amino Acids

Methanol–acetate extraction of the rat cerebral cortex and quantification of 24 amino acids and dipeptides were conducted as described previously [35,79]. Briefly, frozen cerebral cortices were homogenized in ice-cold methanol, then an acetic acid solution was added and the proteins were precipitated. The sodium-citrate buffer system with a Hitachi L-8800 amino acid analyzer was used according to the manufacturer′s user manual, as described previously [35,73].

### 4.8. Data Acquisition and Statistics

Statistical analysis was performed using Statistica 10.0 (StatSoft Inc., Tulsa, OK, USA) and GraphPad Prism, version 7.0 (GraphPad Software Inc., La Jolla, CA, USA). Averaged values are presented as the means ± SEM. Comparisons between multiple experimental groups were performed using one-way ANOVA with Tukey’s post hoc test. Comparison of the two groups was performed using the Mann–Whitney U test or unpaired *t*-test with Welch’s correction. Normal distribution was tested using the Shapiro–Wilk normality test (*p* < 0.05). Differences with *p* < 0.05 were considered significant; differences with 0.05 < *p* < 0.1 were considered trends.

## Figures and Tables

**Table 1:** Influence of acute prenatal hypoxia on the 20th day of gestation (APH-20) on the levels of amino acids and dipeptides in cerebral cortex of the rat offspring. The proteinogenic amino acids are abbreviated by the standard three-letter code. GABA—gamma-aminobutyric acid. Levels of the indicated compounds are presented in μmol per g of the tissue fresh weight as the mean ± SEM. Significance of the differences between the two groups (control and APH-20-exposed) is characterized by the *p*-values according to the Mann–Whitney U test, shown in the table. Significantly different (*p* < 0.05) levels of the compounds are shown in bold, with the corresponding *p*-values colored in red. *n*—The number of rats in each group.

	Females			Males		
	Control *n* = 5	APH-20 *n* = 11	*p*	Control *n* = 6	APH-20 *n* = 8	*p*
**Ala**	0.513 ± 0.030	0.574 ± 0.012	0.786	0.520 ± 0.011	0.534 ± 0.021	0.662
**Anserine**	0.006 ± 0.000	0.017 ± 0.006	0.441	0.011 ± 0.002	0.019 ± 0.008	0.852
**Arg**	0.100 ± 0.010	0.085 ± 0.004	0.071	0.091 ± 0.012	0.102 ± 0.007	0.414
**Asn**	**0.031 ± 0.002**	**0.035 ± 0.002**	0.036	0.035 ± 0.002	0.033 ± 0.002	0.662
**Asp**	2.430 ± 0.220	2.447 ± 0.042	0.393	2.414 ± 0.098	2.372 ± 0.099	0.755
**Carnosine**	**0.012 ± 0.001**	**0.017 ± 0.001**	0.036	0.030 ± 0.007	0.015 ± 0.001	0.181
**Citrulline**	0.024 ± 0.003	0.028 ± 0.002	0.071	0.029 ± 0.002	0.030 ± 0.002	0.662
**Cystine**	0.005 ± 0.001	0.012 ± 0.003	0.571	0.004 ± 0.001	0.011 ± 0.004	0.142
**GABA**	1.270 ± 0.166	1.404 ± 0.034	0.786	1.317 ± 0.023	1.359 ± 0.053	0.573
**Gln**	4.473 ± 0.478	4.596 ± 0.123	0.571	4.003 ± 0.183	4.389 ± 0.248	0.181
**Glu**	**9.783 ± 0.486**	**10.776 ± 0.23**	0.009	9.625 ± 0.237	10.203 ± 0.462	0.282
**Gly**	**0.560 ± 0.031**	**0.639 ± 0.014**	0.000	0.616 ± 0.019	0.604 ± 0.025	0.950
**His**	0.058 ± 0.006	0.061 ± 0.003	0.583	0.055 ± 0.004	0.066 ± 0.005	0.181
**Ile**	0.029 ± 0.005	0.030 ± 0.001	0.913	0.029 ± 0.002	0.030 ± 0.001	0.852
**Leu**	0.058 ± 0.008	0.060 ± 0.002	0.827	0.054 ± 0.003	0.060 ± 0.003	0.081
**Lys**	0.122 ± 0.009	0.144 ± 0.009	0.145	0.114 ± 0.011	0.117 ± 0.008	0.950
**Met**	0.043 ± 0.006	0.038 ± 0.001	0.180	0.043 ± 0.003	0.046 ± 0.003	0.282
**Phe**	**0.035 ± 0.004**	**0.039 ± 0.001**	0.009	**0.033 ± 0.002**	**0.044 ± 0.002**	0.003
**Pro**	0.047 ± 0.008	0.056 ± 0.004	0.221	0.053 ± 0.005	0.063 ± 0.011	0.662
**Ser**	**0.814 ± 0.058**	**0.949 ± 0.025**	0.003	0.818 ± 0.026	0.838 ± 0.039	0.491
**Thr**	**0.431 ± 0.036**	**0.592 ± 0.027**	0.000	**0.389 ± 0.008**	**0.438 ± 0.022**	0.020
**Trp**	**0.035 ± 0.001**	**0.044 ± 0.001**	0.001	**0.025 ± 0.002**	**0.043 ± 0.001**	0.000
**Tyr**	0.056 ± 0.006	0.056 ± 0.005	0.913	0.052 ± 0.003	0.067 ± 0.005	0.108
**Val**	0.077 ± 0.008	0.085 ± 0.002	0.115	0.087 ± 0.003	0.083 ± 0.002	0.345

## Data Availability

The data presented in this study are available in this article (summarized in figures and tables). The raw data are available on request from the corresponding author.

## Abbreviations

APH	acute prenatal hypoxia
CNS	central nervous system
dX	range of RR intervals
ECG	electrocardiogram/electrocardiography
GDH	glutamate dehydrogenase
GOT2	glutamic oxaloacetic transaminase
MDH	malate dehydrogenase
ME	malic enzyme
OGDHC	2-oxoglutarate dehydrogenase complex
PDHC	pyruvate dehydrogenase complex
RMSSD	parasympathetic or relaxation index of the nervous systems state
SI	sympathetic or stress index of the nervous systems state stress index.
